# A Review of the Clinical Utility of Tubule‐Specific Biomarkers in Sickle Cell Nephropathy

**DOI:** 10.1155/bmri/3790001

**Published:** 2026-08-26

**Authors:** Belinda Owusuaa Antwi, Kwame Afriyie Kyei Baffour, Anthony Yaw Dziworshie, Sandy Baffour Adu, Godfred Amoah Appiah

**Affiliations:** ^1^ West African Genetic Medicine Centre, College of Health Sciences, University of Ghana, Legon, Ghana, ug.edu.gh; ^2^ Department of Medical Diagnostics, Kwame Nkrumah University of Science and Technology, Kumasi, Ghana, knust.edu.gh; ^3^ Noguchi Memorial Institute for Medical Research, College of Health Sciences, University of Ghana, Legon, Ghana, ug.edu.gh; ^4^ Department of Biomedical Laboratory Sciences, University for Development Studies, Tamale, Ghana, uds.edu.gh

**Keywords:** early detection, sickle cell disease, sickle cell nephropathy, tubular biomarkers

## Abstract

Sickle cell nephropathy affects about 30%–50% of individuals living with sickle cell disease (SCD), raising the risk of mortality in this population. Traditional renal markers, such as serum creatinine and glomerular filtration rate, fail to detect renal damage until substantial injury occurs. However, growing evidence suggests tubular injury precedes glomerular damage, offering opportunities for earlier intervention through novel biomarkers that detect subclinical renal stress before albuminuria onset. In this narrative review, we evaluated kidney injury molecule‐1, neutrophil gelatinase‐associated lipocalin, N‐acetyl‐*β*‐D‐glucosaminidase, transforming growth factor‐*β*, liver‐type fatty acid binding protein, and monocyte chemoattractant protein‐1 as early indicators of renal damage in individuals with SCD. Additionally, we examine the effect of existing SCD therapy on these biomarkers, highlighting their clinical relevance in sickle cell nephropathy.

## 1. Introduction

Sickle cell disease (SCD) is a group of inherited hematological disorders that affect millions of people worldwide. The pathophysiology originates from a single‐point mutation in the *β*‐globin gene, resulting in the substitution of valine for glutamic acid at the sixth position, which promotes hemoglobin S (HbS) polymerization under deoxygenated conditions [1]. This molecular defect transforms flexible erythrocytes into rigid, sickled cells that adhere to the vascular endothelium, triggering a cascade of endothelial activation mediated by increased expression of vascular cell adhesion molecule‐1 (VCAM‐1) and selectins [2]. The accumulation of sickled erythrocytes and leukocytes on the vessel wall promotes vaso‐occlusion, restricting blood flow and causing tissue ischemia [2]. Over time, these processes lead to chronic hemolysis, multiorgan damage, and premature mortality in SCD patients [3].

The kidneys are highly susceptible to SCD‐related injury because of their unique microvascular structure, high metabolic demand, and sensitivity to hypoxia [4]. Repeated episodes of ischemia‐reperfusion injury, together with chronic hemolysis, endothelial dysfunction, and oxidative stress, progressively damage both the glomerular and tubular compartments of the kidney, ultimately contributing to the development of sickle cell nephropathy (SCN) [2]. Beyond these established mechanisms, emerging experimental evidence suggests that thrombo‐inflammatory mechanisms may contribute to renal injury in sickle cell hemoglobinopathies. Using a humanized mouse model of the sickle cell trait, Trebak et al. [5] demonstrated that activation of coagulation and inflammatory pathways contributes to the development of chronic kidney disease (CKD), further highlighting the complex vascular mechanisms underlying sickle‐associated renal injury.

**Figure 1:**
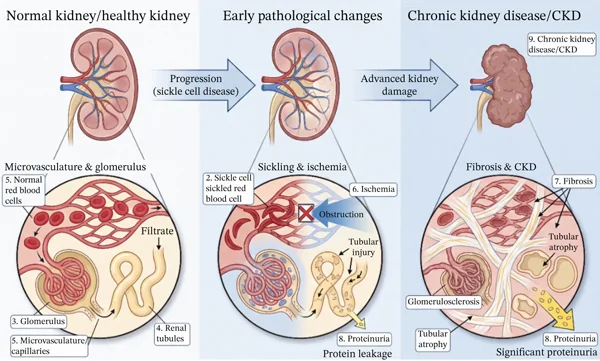
Schematic representation of the pathological progression of sickle cell nephropathy.

The clinical burden of SCN is substantial, with estimates suggesting that 30%–50% of adults with sickle cell anemia develop SCN during their lifetime [6]. Clinically, SCN is characterized by a wide array of renal manifestations, including hyposthenuria, albuminuria, reduced glomerular filtration rate (GFR), hematuria, and eventually, overt proteinuria and CKD [7]. These features often evolve silently in pediatric or even steady‐state SCD populations, making early detection challenging [2]. For instance, a study by Anto et al. [8] documented a CKD prevalence of 39.6% among Ghanaian children aged 5–12 years with SCD (rising to 73.5% in those with the HbSS phenotype), yet serum creatinine was surprisingly lower in HbSS children than in their HbAS counterparts. Similarly, Lekpor et al. [9] demonstrated that circulating biomarkers in 377 children with SCD and the sickle cell trait reported that inflammatory, vascular injury, and neuronal markers correlated with disease severity even in the absence of overt clinical signs. This striking dissociation between CKD burden and creatinine levels illustrates a fundamental limitation of conventional renal markers in SCD. Figure 1 illustrates the pathological progression of SCN.

Emerging evidence indicates that tubular injury often precedes overt glomerular damage, offering a window for intervention [10]. Consequently, there is a growing focus on novel tubular biomarkers that can detect subclinical renal injury before traditional markers like albuminuria or GFR decline become apparent. Among the most studied are kidney injury molecule‐1 (KIM‐1), neutrophil gelatinase‐associated lipocalin (NGAL), and N‐acetyl‐*β*‐D‐glucosaminidase (NAG) [11]. Additional candidates, such as liver‐type fatty acid‐binding protein (L‐FABP), transforming growth factor‐beta (TGF‐*β*), and monocyte chemoattractant protein‐1 (MCP‐1), are also being investigated for their potential roles in SCN pathophysiology [2, 12]. Despite the promise of these biomarkers, challenges remain in their widespread adoption. This narrative review is aimed at providing current evidence on tubule‐specific biomarkers in SCN to accentuate their clinical relevance and applications.

## 2. Methods

### 2.1. Search Strategy and Selection Criteria

We searched electronic databases, including PubMed, Scopus, and Google Scholar, for published research articles from 2006 to 2025, focusing on SCN and early tubular biomarkers. The search strategy was conducted using combinations of the terms “sickle cell disease,” “sickle cell nephropathy,” “tubular biomarkers,” “kidney injury molecule‐1/KIM‐1,” “neutrophil gelatinase‐associated lipocalin/NGAL,” “N‐acetyl‐*β*‐D‐glucosaminidase/NAG,” “transforming growth factor‐*β*/TGF‐*β*,” “liver‐type fatty acid binding protein/L‐FABP,” “monocyte chemoattractant protein‐1/MCP‐1,” and “early kidney injury.” Additional relevant articles were identified through reference‐list screening.

### 2.2. Eligibility Criteria

Studies that examined one or more tubular biomarkers in SCN, addressed early kidney injury or tubular dysfunction, or provided a clinically relevant context for biomarker interpretation were included. Clinical studies, including cross‐sectional, cohort, and longitudinal designs, were considered, along with experimental studies and selected review articles for background context. Studies not directly related to SCD‐associated renal outcomes or not assessing any biomarker of interest were excluded. Only publications available in English were considered.

### 2.3. Approach to Synthesis

Findings were synthesized descriptively, with attention to the phase of nephropathy in which each biomarker was studied, the clinical context of measurement, and the distinction between acute injury during vaso‐occlusive crises (VOC) and chronic subclinical tubular stress in steady‐state disease.

## 3. Discussion

### 3.1. Current Evidence of Tubular Biomarkers Across Phases of SCN

In the past decade, there has been increasing interest in a group of urinary biomarkers that are released or upregulated in response to tubular cell damage [13]. In the acute phase, typically coinciding with VOC or ischemia‐reperfusion events, biomarkers reflect rapid tubular epithelial injury and predict short‐term outcomes such as acute kidney injury (AKI) and mortality. In the chronic phase, these biomarkers signal progressive tubular stress that precedes albuminuria and CKD. Evidence for each biomarker is therefore reviewed with explicit attention to the disease phase in which it was studied. These biomarkers can detect nephron injury from multiple angles: ischemia, inflammation, lysosomal disruption, and oxidative stress [14]. Despite variations in methodology and geographic focus, these biomarkers have consistently indicated early kidney damage in children and asymptomatic SCD patients [15]. Although earlier studies primarily focused on adults with proteinuria or chronic renal abnormalities, more recent investigations have increasingly evaluated pediatric and asymptomatic populations to identify subclinical tubular injury before overt nephropathy develops [13, 16]. Thus, these findings suggest that tubular biomarkers may have utility across the lifespan of patients with SCD, although their diagnostic performance and clinical interpretation may vary depending on age , disease severity, and clinical setting (Table 1). Their application in clinical practice could therefore transform the current paradigm of renal health surveillance from reactive to proactive [15].

**Table 1:** Studies of tubular biomarkers in SCN, organized by study design, population, phase of disease, key outcomes, and clinical relevance.

Biomarker(s)	Source(s)	Study population	Study design	Phase of SCN	Key outcomes	Clinical relevance/interpretation
NAG	[17]	Adults with HbS/*β*‐thalassemia	Cross‐sectional	Chronic	NAG was significantly elevated versus controls and associated with proteinuria and early tubular dysfunction despite normal creatinine.	First evidence supporting tubular injury in SCD‐spectrum disease; useful for early chronic tubular stress.
KIM‐1	[18]	Multicenter longitudinal cohort of SCD patients	Longitudinal/cohort	Chronic	Higher KIM‐1 levels were associated with the progression of albuminuria over time. Albuminuria progression and renal decline were also linked to sex, with faster eGFR decline in males reported in the broader cohort literature.	Supports KIM‐1 as a progression marker in renal trajectory.
KIM‐1, NAG, NGAL	[19]	SCD patients (children and adults)	Cross‐sectional	Chronic	KIM‐1 and NAG were strongly associated with albuminuria, whereas NGAL showed a weaker association in steady‐state SCD.	KIM‐1 and NAG appear more relevant than NGAL in chronic disease.
NGAL	[20]	Children with steady‐state SCD	Cross‐sectional	Chronic	Elevated NGAL was associated with early renal dysfunction and albuminuria in clinically stable SCD children.	Supports NGAL as a marker of subclinical chronic renal injury.
NGAL	[21]	Hospitalized children with SCD and AKI	Prospective cohort	Acute	NGAL was markedly elevated in AKI and was a strong predictor of AKI severity and mortality.	Strong evidence for NGAL in acute ischemic or vaso‐occlusive kidney injury.
KIM‐1, NAG, NGAL	[22]	Adults with SCD	Cross‐sectional	Chronic	A multibiomarker panel improved early CKD detection. KIM‐1 and NAG were stronger tubular injury markers than NGAL.	Supports a panel‐based approach for chronic kidney disease detection.
NGAL, NAG	[15]	SCD patients with mixed severity.	Observational	Mixed/unclear	Early elevation of NGAL and NAG detected subclinical tubular injury before overt nephropathy.	Suggests that both markers may detect early renal stress across disease stages.
TGF‐*β*	[23, 24]	SCD and SCN populations,	Observational	Chronic	Elevated TGF‐*β* was linked to glomerulosclerosis, interstitial fibrosis, and progressive renal scarring.	Better viewed as a marker of disease progression and fibrosis than early tubular injury.
L‐FABP	[25–28]	Adult and pediatric renal injury cohorts	Observational	Chronic, possibly early injury	Urinary L‐FABP increased with ischemia, hypoxia, and oxidative stress and may precede albuminuria.	Promising adjunct biomarker for oxidative stress–mediated tubular injury, but validation in SCD remains limited.
MCP‐1	[29]	Children with SCA with/without albuminuria	Cross‐sectional	Chronic	Children with albuminuria had significantly higher urinary cytokines and chemokines levels; albumin/creatinine ratio positively correlated with MCP‐1.	Indicates that inflammation is strongly associated with albuminuria; urinary MCP‐1 reflects renal injury.
MCP‐1	[12]	SCA patients stratified by HU usage	Prospective cohort	Chronic	MCP‐1 was higher in SCA patients who were not on HU.	Correlates with renal damage associated with oxidative stress.

Abbreviations: AKI, acute kidney injury; CKD, chronic kidney disease; HbS, hemoglobin S; HU, hydroxyurea; KIM‐1, kidney injury molecule‐1; L‐FABP, liver‐type fatty acid binding protein; MCP‐1, monocyte chemoattractant protein‐1; NAG, N‐acetyl‐*β*‐D‐glucosaminidase; NGAL, neutrophil gelatinase‐associated lipocalin; SCD, sickle cell disease; SCN, sickle cell nephropathy; TGF‐*β*, transforming growth factor‐beta.

### 3.2. KIM‐1

KIM‐1 is a transmembrane glycoprotein that becomes highly expressed on the surface of proximal tubular epithelial cells following kidney damage [30]. The coding gene, Hepatitis A virus cellular receptor 1 (HAVCR1), shows minimal or undetectable expression under physiological conditions but is markedly upregulated after ischemic or nephrotoxic insult [31]. Once expressed, it doubles as a scavenger receptor that facilitates the removal of apoptotic debris in the tubular lumen, a mechanism essential for postinjury epithelial recovery [31]. Beyond its role in tubular repair, KIM‐1 is also thought to be a marker for neutrophil antibody‐associated vasculitis in glomerulonephritis and tubulointerstitial injury [32].

KIM‐1 urinary concentration has been shown to increase before detectable changes in serum creatinine or estimated GFR, making it highly useful in early‐stage diagnosis of tubular injury [33]. Sundaram et al. [19], in a mixed cross‐sectional study of children and adults with SCD, demonstrated that urinary KIM‐1 levels were significantly elevated in SCD patients with microalbuminuria and macroalbuminuria compared with those without albuminuria. These findings suggest that KIM‐1 reflects the presence and severity of early renal injury in SCD. Beyond its diagnostic value, accumulating longitudinal evidence indicates that KIM‐1 may also have prognostic utility. Niss et al. [18], in a multicenter longitudinal study involving predominantly female participants (54%) across 11 study sites in the United States and Jamaica from 2009 to 2017, found that higher KIM‐1 levels were associated with progression of albuminuria over time in patients with SCD. More recently, Ataga et al. [34] further demonstrated that KIM‐1, together with other novel biomarkers, was associated with persistent albuminuria and CKD progression in sickle cell anemia, supporting the use of biomarker panels rather than single biomarkers for predicting disease progression. Consistent with these clinical observations, KIM‐1 has also shown good correlation with histological features of tubulointerstitial injury and chronic fibrosis in both animal and human models, supporting its potential as a prognostic marker for long‐term renal outcomes [35]. Nonetheless, the performance of KIM‐1 in CKD is variable across studies, and its role in chronic SCN remains less well defined.

### 3.3. NAG

NAG is a lysosomal enzyme released from proximal tubular cells [36]. There are three *β*‐*N*‐acetylhexosaminidase isozymes: Hex A (comprising one *α*‐subunit and one *β*‐subunit), Hex B (two *β*‐subunits), and minor Hex S (two *α*‐subunits) in human lysosomes [37]. NAG normally remains intracellular due to its large molecular size (130 kDa), which prevents it from passing through the glomerular filtration barrier [38]. As a result, its appearance in urine is therefore a reliable indicator of proximal tubular cell damage or lysosomal stress [22]. Chronic hemolysis and repeated ischemia‐reperfusion injuries also exacerbate oxidative stress in the renal tubular environment, triggering lysosomal activation and NAG release [39]. Elevated urinary NAG levels have been documented in SCD patients, even in the absence of overt proteinuria, reinforcing its utility for early detection [13]. Unlike the other markers, NAG is considered highly specific for tubular damage and less affected by transient hemodynamic changes or systemic inflammation [13]. Its persistent elevation, even in patients with stable GFR but with early renal involvement, makes it a suitable adjunct to albuminuria and serum creatinine in comprehensive renal assessments [38]. However, its diagnostic use may be influenced by factors such as age, fever, and urinary tract infections, which can also elevate NAG excretion [36]. Therefore, the interpretation of urinary NAG levels should be contextualized within the clinical setting and, ideally, confirmed by additional biomarkers [40].

### 3.4. NGAL

NGAL is an iron transport protein expressed in various tissues and encoded by the lipocalin‐2 (LCN2) gene on chromosome 9 [41]. It is released by tubular epithelial cells in response to ischemic and nephrotoxic insults and rises rapidly in urine, often within 2–6 h after kidney injury [41, 42]. Beyond injury responses, NGAL modulates iron homeostasis by binding siderophores, limiting iron availability to bacteria and influencing oxidative stress; processes highly relevant in the hemolytic environment of SCD [20, 42]. This dual role in iron regulation and renal injury strengthens the biological rationale for its elevation in SCD, especially during VOCs when hemolysis and inflammation are intensified [20, 21].

In clinical practice, NGAL′s evidence is prominent across acute and chronic SCD populations. In children with SCD, elevated urine NGAL reliably predicts AKI and mortality risk [20]. In the chronic phase, persistently elevated NGAL correlates with declining GFR and structural kidney damage, even when albuminuria is absent [21]. This utility is further supported by recent data. A multicenter case‐control study in Ghana evaluated steady‐state SCD patients and reported significantly higher serum NGAL levels in those with microalbuminuria compared with those without; NGAL showed excellent diagnostic performance for kidney disease, with a sensitivity of 91.2% and a specificity of 74.7%. The authors noted that NGAL outperformed conventional renal markers such as serum creatinine and urea as an early indicator of kidney disease, underscoring its clinical value in the early detection of SCN [43]. Despite this promise, NGAL lacks complete renal specificity because levels can also rise in response to systemic infections, inflammation, or malignancies [21]. For this reason, its diagnostic accuracy improves when used alongside more renal‐specific markers like KIM‐1 or NAG [19].

### 3.5. TGF‐*β*

The TGF‐*β* is a pleiotropic cytokine that plays a major role in renal fibrosis and CKD. It is involved in regulating extracellular matrix production, mesangial expansion, and fibroblast activation, thereby contributing to structural remodeling of the renal tissue [44]. In SCD, chronic hemolysis, oxidative stress, and repeated ischemia‐reperfusion injury create a proinflammatory environment that can upregulate TGF‐*β* signaling pathways, promoting progressive renal scarring [23]. Elevated levels of TGF‐*β* have been associated with glomerulosclerosis and interstitial fibrosis, suggesting that it reflects late‐stage or progressive renal disease rather than early tubular dysfunction [24]. Although studies in SCN are relatively limited, available evidence suggests that increased TGF‐*β* expression correlates with worsening renal pathology and may contribute to the transition from functional abnormalities to irreversible structural damage [24]. As such, TGF‐*β* is better viewed as a marker of disease progression rather than an early diagnostic marker.

### 3.6. L‐FABP

The L‐FABP is expressed in proximal tubular epithelial cells and plays a role in intracellular fatty acid transport and protection against oxidative stress. Under ischemic, hypoxic, or oxidative burden, L‐FABP is released into urine, making it a potential marker for early tubular injury [25]. Studies in both adult and pediatric populations have shown that elevated urinary L‐FABP correlates with early tubular dysfunction and may precede overt albuminuria, suggesting a role in detecting subclinical kidney injury [26]. Compared with NGAL, L‐FABP may offer greater specificity for oxidative stress–mediated tubular injury, which is highly relevant in the pathophysiology of SCN [27]. However, despite its potential, its diagnostic performance has been variable across studies, with meta‐analyses reporting moderate sensitivity and specificity and highlighting the need for further validation in diverse clinical settings [28].

### 3.7. Effects of Sickle Cell Therapies on Renal Outcomes and Tubular Biomarkers

Therapeutic strategies for SCD have evolved over the last few decades, moving beyond supportive care to include targeted disease‐modifying interventions [45]. Although these treatments are aimed primarily at reducing hemolysis, VOC, and chronic organ damage, several may also influence the onset and progression of SCN and impact the release of tubular biomarkers.

#### 3.7.1. Hydroxyurea (HU)

**Figure 2:**
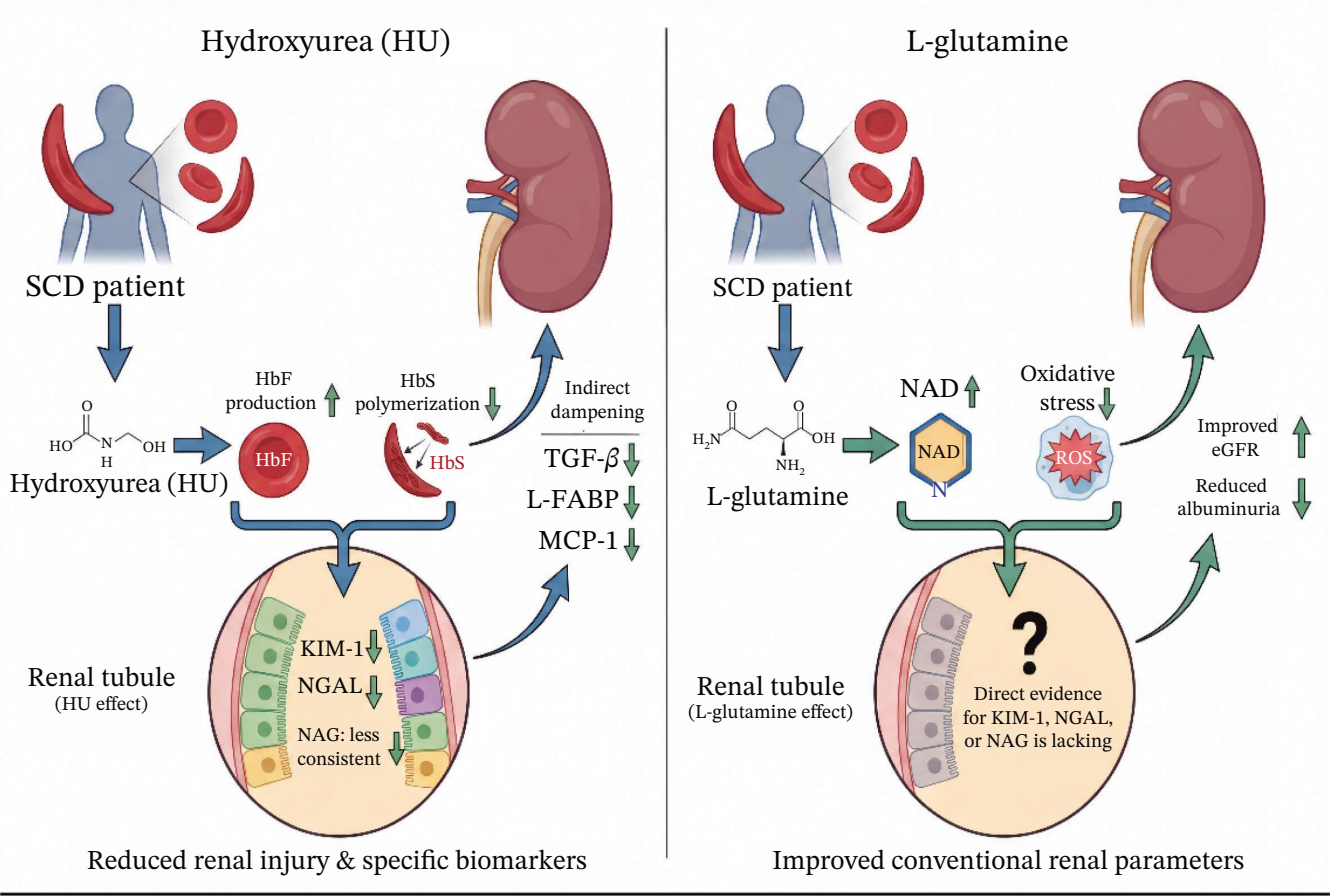
Effects of hydroxyurea and L‐glutamine on renal outcomes and tubular biomarkers.

HU remains the most established disease‐modifying therapy for SCD, with decades of evidence supporting its efficacy [45]. It acts primarily by increasing HbF production, which inhibits HbS polymerization [46]. Reported HU′s effect on tubular biomarkers includes reductions in urinary KIM‐1 and NGAL in HU‐treated cohorts compared with untreated cohorts [47]. At the preclinical level, a study using Townes humanized sickle cell mice demonstrated that HU significantly reduced NGAL and improved renal histopathology, with evidence of reduced vascular congestion, glomerulosclerosis, and tubular damage [47]. These preclinical findings were further supported by earlier ischemia‐reperfusion injury models, in which HU significantly lowered serum creatinine, NGAL, and KIM‐1 levels. Multiple human cohort studies have documented significantly lower urinary KIM‐1 concentrations in HU‐treated patients compared with untreated SCD patients. These reductions corresponded to lower albuminuria and less advanced nephropathy staging in treated cohorts [48], confirming that the change in biomarker level reflects genuine attenuation of tubular injury rather than an effect on the assay itself. Thus, these studies provide biological plausibility for HU′s reno‐protective effects at the tubular level, though direct measurement of urinary KIM‐1, NGAL, and NAG in treated human SCD cohorts remains limited. Also, the evidence for HU′s effect on urinary NAG is less consistent than for KIM‐1 and NGAL. Reports from multiple cohort studies show variable outcomes: Some studies report modest reductions in NAG activity in HU‐treated patients, whereas others find no statistically significant difference compared with untreated controls [47]. This variability may reflect differences in disease stage at study enrolment, duration and dose of HU therapy, achieved HbF levels, and the extent of pre‐existing tubular injury across study populations. The evidence for HU′s effect on TGF‐*β*, L‐FABP, and MCP‐1 is comparatively limited, though mechanistically plausible. HU′s capacity to attenuate hemolysis, vaso‐occlusion, and the resultant proinflammatory milieu suggests an indirect dampening of the upstream stimuli that drive TGF‐*β* upregulation and progressive renal scarring [45]. One pediatric study measuring urinary TGF‐*β*1 alongside NGAL in SCD patients included a subset receiving HU therapy, but the effect of HU on TGF‐*β*1 levels was not assessed as a primary outcome [49]. No major clinical trial has measured L‐FABP as a primary renal endpoint in HU‐treated SCD cohorts. However, given that L‐FABP is released from proximal tubular epithelial cells in response to oxidative stress and hypoxia, HU′s well‐documented antioxidant properties provide an indirect mechanistic rationale for its potential attenuation [25]. Studies have demonstrated that HU therapy is associated with a reduction in systemic oxidative stress markers in SCA patients [50], and preclinical data confirm that HU reduces plasma heme, reactive oxygen species (ROS), and proinflammatory cytokines, which are the same mediators that drive proximal tubular L‐FABP and MCP‐1 activation and release [51] (Figure 2).

#### 3.7.2. L‐Glutamine

L‐glutamine was approved in 2017 as a supportive therapy for SCD based on its ability to reduce VOC frequency [52]. Its mechanism involves increasing the availability of nicotinamide adenine dinucleotide (NAD) in red blood cells, which helps reduce oxidative stress and improve red cell membrane integrity [52]. Although oxidative stress reduction could theoretically protect renal tubular epithelial cells and indirectly lower biomarker levels [53], there are no currently published clinical trials directly assessing L‐glutamine′s effect on KIM‐1, NGAL, NAG, or MCP‐1 in SCD populations. The most relevant human data on renal outcomes under L‐glutamine comes from an observational study that followed 10 patients (4 pediatric patients and 6 adults) with confirmed HbSS genotype receiving oral L‐glutamine at approximately 0.3 g/kg twice daily for 120 weeks. Renal function was assessed at baseline, 48, and 120 weeks using eGFR, serum creatinine, and albuminuria. The study reported sustained improvements in eGFR and reductions in albuminuria at both time points, alongside decreases in VOC frequency and hemolysis markers, including lactate dehydrogenase [54]. These findings indicate that L‐glutamine is associated with improvements in conventional glomerular‐level renal parameters over an extended follow‐up period. However, the study did not measure KIM‐1, NGAL, or NAG, and therefore does not establish whether the observed improvement in eGFR and albuminuria was accompanied by any change in tubular injury (Figure 2).

### 3.8. Diagnostic Relevance of Early Tubular Biomarkers in SCD

Early detection of renal impairment in individuals with SCD is essential to identify acute tubular injury during VOC to prevent progression to AKI and to detect subclinical chronic tubular stress early enough to delay or prevent CKD and end‐stage renal disease [55]. KIM‐1, NGAL, NAG, TGF‐*β*, L‐FABP, and MCP‐1 offer promise for identifying subclinical renal injury before conventional indicators, such as serum creatinine or estimated GFR, become abnormal [56]. Although each biomarker reflects aspects of tubular injury and repair, their combined interpretation provides a more comprehensive and nuanced understanding of renal pathology in SCD and may improve early identification of individuals at risk of progressive SCN. However, biomarker interpretation should not be considered in isolation, as SCN progression is influenced by multiple patient‐specific factors, including genetic and clinical risk determinants. Recent longitudinal evidence from Sun et al. [57] identified diabetes mellitus and high‐risk apolipoprotein L1 (APOL1) genotypes as independent predictors of CKD progression in individuals with SCD. These findings highlight the importance of integrating tubular biomarkers with established risk factors to improve patient stratification, identify individuals requiring closer renal surveillance, and guide earlier therapeutic intervention.

An additional consideration in biomarker interpretation is the influence of biological factors, particularly sex, on renal disease progression in SCD. Sex‐related differences in renal trajectories have been demonstrated in both experimental and clinical studies, suggesting that biomarker thresholds may require sex‐specific consideration. In humanized HbSS mouse models, male mice exhibited early hyperfiltration followed by progressive decline in GFR, whereas female mice demonstrated a prolonged but attenuated hyperfiltration phase with delayed renal injury [58]. These findings suggest that females may enter the chronic injury phase later but are not protected from disease progression. Similar patterns have been reported in multicenter human cohort studies [59]. Consequently, males may demonstrate apparently preserved GFR despite ongoing tubular injury and nephron loss, a stage during which tubular biomarkers may increase before detectable changes in creatinine or eGFR occur [59]. Supporting this, sex was independently associated with albuminuria in multivariate analysis alongside older age, anemia, higher bilirubin levels, and KIM‐1 concentrations [18].

Beyond improving early detection, these biomarkers have important implications for clinical monitoring, particularly in low‐resource healthcare settings where access to advanced imaging techniques and specialist nephrology services may be limited [11]. Their ability to detect early renal damage through noninvasive urine testing makes them suitable for screening and monitoring at the primary care level [18]. Standardizing these assays into cost‐effective, point‐of‐care platforms could further expand their use in community‐based surveillance and long‐term disease management [60].

Despite their diagnostic potential, several challenges currently limit their widespread clinical adoption for these tubular biomarkers. Tests for KIM‐1, NGAL, NAG, TGF‐*β*, L‐FABP, and MCP‐1 remain largely confined to research settings, and assay costs may restrict availability in resource‐limited regions [11]. Additionally, these biomarkers are not entirely specific to SCD‐related renal injury; elevated levels may occur in other renal or systemic conditions, creating diagnostic uncertainty [32]. Longitudinal studies linking biomarker fluctuations with long‐term renal outcomes in SCD remain limited [18]. Future research should therefore focus on establishing standardized assays, validating biomarker thresholds across diverse populations, and developing sex‐specific reference ranges that account for differences in renal disease progression. Addressing these gaps will be essential for integrating tubular biomarkers into routine clinical care and maximizing their potential for early SCN detection and intervention.

## 4. Conclusion

Renal complications remain a significant cause of morbidity among individuals with SCD, arising through both acute tubular injury during VOC and chronic subclinical tubular stress preceding CKD. Tubular biomarkers such as KIM‐1, NGAL, NAG, TGF‐*β*, L‐FABP, and MCP‐1 demonstrate strong potential for detecting injury across both phases. Their combined application may enhance early diagnosis and support timely therapeutic interventions. Nonetheless, broader clinical adoption is constrained by assay cost, limited availability, and the need for further validation. Advancing standardization, developing phase‐specific reference ranges for each sex, and integrating these markers into clinical workflows could strengthen both SCN management and early CKD screening in SCD.

## Author Contributions

B.O.A. conceived the original idea for this review with input from G.A.A. B.O.A., K.A.K.B., A.Y.D., and S.B.A. prepared the original draft, which was reviewed and edited by G.A.A. and B.O.A. G.A.A. provided oversight and leadership.

## Funding

No funding was received for this manuscript.

## Disclosure

All authors read and approved the final manuscript.

## Conflicts of Interest

The authors declare no conflicts of interest.

## Data Availability

No new data were created or analyzed in this study.
